# PKR involvement in Alzheimer’s disease

**DOI:** 10.1186/s13195-017-0308-0

**Published:** 2017-10-05

**Authors:** Jacques Hugon, François Mouton-Liger, Julien Dumurgier, Claire Paquet

**Affiliations:** 1 Center of Cognitive Neurology and Inserm U942 Lariboisière Hospital AP-HP University Paris Diderot, 75010 Paris, France; 2 Inserm U1127 Institut du Cerveau et de la Moelle, 75013 Paris, France; 3 Center of Cognitive Neurology, Lariboisière FW Hospital, 200 rue du Faubourg Saint Denis, 75010 Paris, France

**Keywords:** Alzheimer’s disease, PKR, Kinases, Amyloid, Neurons, Apoptosis, Biomarkers, Therapeutics

## Abstract

**Background:**

Brain lesions in Alzheimer’s disease (AD) are characterized by Aβ accumulation, neurofibrillary tangles, and synaptic and neuronal vanishing. According to the amyloid cascade hypothesis, Aβ1-42 oligomers could trigger a neurotoxic cascade with kinase activation that leads to tau phosphorylation and neurodegeneration. Detrimental pathways that are associated with kinase activation could also be linked to the triggering of direct neuronal death, the production of free radicals, and neuroinflammation.

**Results:**

Among these kinases, PKR (eukaryotic initiation factor 2α kinase 2) is a pro-apoptotic enzyme that inhibits translation and that has been implicated in several molecular pathways that lead to AD brain lesions and disturbed memory formation. PKR accumulates in degenerating neurons and is activated by Aβ1-42 neurotoxicity. It might modulate Aβ synthesis through BACE 1 induction. PKR is increased in cerebrospinal fluid from patients with AD and mild cognitive impairment and can induce the activation of pro-inflammatory pathways leading to TNFα and IL1-β production. In addition, experimentally, PKR seems to down-regulate the molecular processes of memory consolidation. This review highlights the major findings linking PKR and abnormal brain metabolism associated with AD lesions.

**Conclusions:**

Studying the detrimental role of PKR signaling in AD could pave the way for a neuroprotective strategy in which PKR inhibition could reduce neuronal demise and alleviate cognitive decline as well as the cumbersome burden of AD for patients.

## Background

With aging populations, Alzheimer’s disease (AD) has become a major public health problem in developed countries [1]. The pathology of AD involves senile plaques made of accumulated Aβ peptide, neurofibrillary tangles with abnormally phosphorylated tau protein, and synaptic and neuronal losses [2]. The cause of AD is not known, but the Aβ peptide could be toxic according to the “amyloid cascade hypothesis” [3]. Aβ is formed after the cleavage of amyloid precursor protein (APP) by β secretase (BACE1) and γ secretase. The amyloid cascade hypothesis proposes that the accumulation of Aβ or its oligomeric forms could be responsible for deleterious consequences, including neuronal and synaptic demise, as well as dementia. The cause of Aβ accumulation in sporadic forms of AD is not fully understood but it might be linked to increased Aβ production due to enhanced BACE1 and γ secretase activities or to reduced Aβ degradation [4]. Many pathogenic mechanisms have been assessed in AD, and the role of kinases has often been linked to tau phosphorylation [5, 6]. However, few studies have explored the links between kinase activation and other brain lesions [7]. This review analyzes recent reports implicating the kinase PKR in the pathogenesis of the neuronal degeneration observed in AD (Table 1).

**Table 1:** Published reports on PKR and Alzheimer’s disease

Report	Sample	Results
Chang et al. 2002 [45]	Brain	pPKR neuronal accumulation
Peel et al. 2003 [39]	Brain	pPKR neuronal accumulation
Onuki et al. 2004 [32]	Brain	pPKR neuronal accumulation
Paccalin et al. 2006 [71]	PBL	pPKR increased level
Page et al. 2006 [40]	Brain	pPKR increased concentration
Bullido et al. 2008 [74]	DNA	PKR gene association
Damjanac et al. 2009 [72]	PBL	PKR-dependent increases in P53, Redd1
Couturier et al. 2010 [35]	PBL	PKR control of inflammation
Bose et al. 2011 [47]	Brain	Co-localization of pPKR and ptau
Paquet et al. 2012 [69]	Brain	Increased levels of PKR activator PACT
Mouton-Liger et al. 2012 [76]	CSF	Increased levels of PKR and pPKR
Badia et al. 2013 [73]	PBL	Increased levels of PKR RNA in ApoE4 patients
Dumurgier et al. 2013 [77]	CSF	CSF pPKR predicts cognitive decline
Paquet et al. 2015 [79]	Brain	Aβ vaccine reduces pPKR load
Taga et al. 2017 [70]	Brain	Correlations cognitive scores and pPKR load

Non-exhaustive list of published studies that assessed the levels of PKR signals in human AD samples, including brain, peripheral blood lymphocytes (*PBL*), and cerebrospinal fluid (*CSF*)

PKR is a ubiquitous 551 amino acid protein associated with two protein families, the eIF2α-kinase family and the dsRNA-binding protein family [8]. It has a serine/threonine kinase domain situated at the C-terminal region and was identified as a protein kinase that is activated by double-stranded RNA and plays a major role in the defense against viruses [9]. It is a stress and pro-apoptotic kinase that can be activated by interferons, TNFα, endoplasmic reticulum (ER) stress, reactive oxygen species (ROS), and calcium [8]. PKR can block protein synthesis by phosphorylation of the eukaryotic translation initiation factor 2α (eIF2α), which ultimately decreases or prevents viral replication. It is activated through a complex mechanism that combines displacement of an N-terminal inhibitory domain, dimerization, and autophosphorylation of the activation loop on two residues (Thr446 and Thr451). Phosphorylation at the Thr446 site occurs before the phosphorylation of Thr451. The PKR activator protein PACT can activate PKR in the absence of double-stranded RNA and during cell stress. PKR can bind to cellular proteins and participate in several complexes through protein–protein interactions (for a review on PKR see Garcia et al. [8]).

## Integrated stress response

**Fig. 1:**
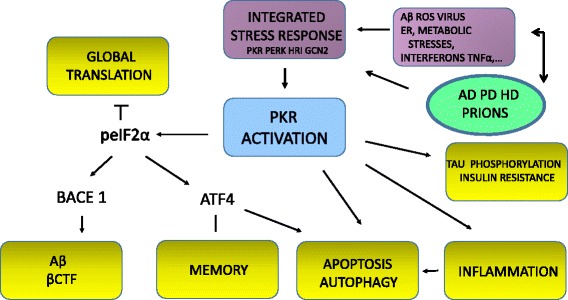
The possible signaling stress pathways contributing to the integrated stress response and PKR activation in neurodegenerative diseases, as well as the molecular consequences of PKR activation in AD, Parkinson’s disease (*PD*) and Huntington’s disease (*HD*)

Various stresses can be induced, and eukaryotic cells have an adaptive response called the integrated stress response (ISR) that can restore cell homeostasis [10]. The main molecular event in this response is the phosphorylation of eIF2α, which can lead to a global reduction in translation and the induction of selected genes, including the transcription factors ATF4 and BACE 1. Four eIF2 kinases catalyze this phosphorylation, PKR, PERK, GCN2, and HRI, which are all induced by specific or common stresses. The ISR is primarily a pro-survival pathway, but prolonged stress can lead to cell death. ATF4 has been implicated in cell survival or apoptosis and memory formation. Conflicting results have been proposed by different groups, showing either a constraint of memory [11] or that ATF4 is a key physiological regulator of memory [12]. In addition to PKR, PERK has also been implicated in neurodegeneration through the induction of the unfolded protein response (UPR) [13]. The UPR is a protective reaction triggered by the occurrence of ER stress that decreases the unfolded protein load and assures a normal protein‐folding process [14]. Prolonged UPR might be detrimental to cell survival. PERK is also a new therapeutic target in AD [15]. ISR is depicted schematically in Fig. 1. Converging cellular stresses, such as ER stress, can concurrently activate PKR and PERK. The question that should be addressed is what involvement PKR has in the genesis of lesions, including neuronal apoptosis and autophagy, neuroinflammation, and Aβ formation and toxicity. This review focuses on the participation of this eIF2α kinase in the development of abnormal signaling pathways associated with neurodegeneration and memory disturbances.

## PKR in apoptosis and autophagy

Many reports have shown that PKR is a pro-apoptotic kinase in various cells, including in neurons. For example, in cultures of retinal ganglion neurons, tunicamycin exposure induced ER stress, PKR activation, and widespread neuronal apoptosis. The pretreatment of cell cultures with the PKR inhibitor C16 (also designated PKRi) or with PKR siRNA attenuated neuronal or retinal ganglion cell death induced by tunicamycin [16, 17]. The authors concluded that inhibiting PKR activation is neuroprotective. In 2007, data from mixed cortical cultures revealed that the toxic protein GP-120 of the human immunodeficiency virus 1 increased PKR phosphorylation and caspase 3 activation. The pharmacological pretreatment of cultures with two PKR inhibitors reduced neuronal apoptosis [18]. The exposure of human neuroblastoma cells SH-SY5Y to interferon-β induced the activation of PKR and caspase 3 cleavage, and this effect was inhibited by the PKR inhibitor C16 [19]. In 2014, a report demonstrated that acute striatal injection of the excitotoxic compound quinolinic acid locally produced PKR activation neuroinflammation and neuronal apoptosis in vivo. An intraperitoneal injection of the PKR inhibitor C16 reduced PKR activation IL-1β levels and neuronal apoptosis [20]. PKR is also involved in the induction of autophagy, and this process could be linked to the protein STAT 3, which directly interacts with PKR [21]. In a recent study, Bordi et al. [22] have demonstrated that abnormal autophagy could contribute to the pathogenesis of AD lesions. Increased triggering of autophagy associated with reduced lysosomal clearance of substrates could lead to autophagic pathology and neuritic dystrophy detected in AD. Further research will be needed to determine whether PKR can participate in this autophagic induction of neurodegeneration.

## PKR and inflammation

Previous reports have demonstrated that PKR is an active player in innate immunity and could participate in several inflammatory pathways [23]. Published data have shown that PKR is involved via an interaction with NLRP3 in HMGB1 release and IL-1β production [24, 25], although these findings are still being debated [26]. In addition, PKR can trigger the NF Kappaβ pathway necessary for TNFα expression via direct protein interactions with I Kappaβ [27]. Finally, PKR can interact with the MAPK pathways and can trigger the activation of JNK and P38 kinases, which are also implicated in neuronal death and inflammation [28]. In conclusion, PKR is activated during three detrimental cellular events, that is, apoptosis, autophagy, and inflammation, which are prominent features of AD brain lesions. Future studies will be necessary to determine the exact starting time of brain PKR activation in the long evolution of preclinical AD brain lesions.

## PKR in Aβ metabolism and neurotoxicity

In vitro studies have revealed that PKR is activated by Aβ peptide toxicity. In 2002, a report showed that, in a human neuroblastoma cell line and in primary neuronal cultures, Aβ exposure induced PKR activation, eIF2α phosphorylation, and apoptosis. The use of dominant-negative PKR cell lines or PKR knockout neurons and calcium blockers reduced the levels of neuronal apoptosis, which suggested that PKR could be involved in calcium-mediated Aβ neurotoxicity [29]. Further studies have revealed that caspase 3 could modulate PKR activation and apoptosis [30, 31]. Using a randomized ribozyme library, the authors found that PKR was involved and activated in ER stress induced by tunicamycin in human neuroblastoma cells [32]. Surprisingly, another report did not detect UPR activation in cultured neurons exposed to Aβ, whereas PKR was clearly activated [31]. Tunicamycin exposure has also been explored in human neuroblastoma cells, and the results have shown that the PKR inhibitor C16 or the overexpression of a dominant-negative PKR attenuates neural cell apoptosis [17, 33]. Recently, it was shown in primary neuronal cultures from wild-type and PKR knockout mice that Aβ toxicity was blocked by genetic deletion of PKR and the JNK inhibitor XG 102, suggesting that dual kinase inhibition might be efficient for enhanced neuroprotection [34]. In addition, it was demonstrated that the PKR inhibitor C16 reduces the release of the inflammatory cytokines TNFα and IL-1β in mixed co-cultures of neurons and microglial cells [35]. PKR can control the levels of BACE 1 protein in human neuroblastoma cells exposed to oxidative stress, which suggests that PKR could modulate Aβ production [36]. In addition, the increased activity of BACE1 could also lead to synthesis of the β-cleaved carboxy-terminal fragment of APP (βCTF), which can recruit APPL to rab5 endosomes and can abnormally increase endocytosis and impair axonal transport [37]. Overall, PKR inhibition reduces Aβ-induced apoptosis neuroinflammation and BACE1 levels in cell cultures.

Reports in experimental animals have confirmed the outcomes of the in vitro studies. In 2003, the first histological studies showing that activated PKR was detected in the brain of APP/PS1 knock-in mice were published, and PKR was observed around plaques and in dystrophic neurites [38, 39]. Data from immunochemical and histological methods confirmed the results in APP/PS1 knock-in mice, which demonstrated that phosphorylated PKR was increased in the brain of mutated mice and was mostly located in hippocampal degenerating neurons. Activated PKR was found in the cytoplasm and nucleus, as well as co-localized with neuronal apoptotic markers [40]. A more recent study showed that, in various experimental models, including monkeys, intra-cerebroventricular injection of Aβ oligomers induced PKR and eIF2α phosphorylation along with cognitive deficits via a mechanism linked to TNFα production. These effects were abolished in PKR and TNFα knockout mice, which suggested that PKR activation was linked to a TNFα-mediated process induced by Aβ oligomer neurotoxicity [41]. Two studies have shown that PKR can be implicated in brain Aβ production in wild-type mice exposed to thiamine deficiency or to peripheral inflammation following systemic lipopolysaccharide administration. Both effects on Aβ levels were reversed in PKR knockout mice [42, 43]. In addition, the pharmacological inhibition of PKR with the compound C16 transiently prevented neuroinflammation in APPswePS1dEç transgenic mice, but surprisingly increased Aβ load at 18 months of age in treated mice compared with untreated mice [44]. The models used in these various studies are different, and the mechanisms of PKR inhibition and further studies are certainly needed to establish the long-term effects of PKR inhibition on Aβ production in experimental models of neurodegeneration.

## PKR and tau phosphorylation

It was demonstrated in early reports on AD human brains [39, 45] that phosphorylated PKR could co-localize with phosphorylated tau in affected neurons. The question raised by these findings was: could PKR directly or indirectly participate in tau phosphorylation? Two studies have addressed this subject. The first report demonstrated that, in rat neuronal cultures, the phosphatase inhibitor okadaic acid can induce tau and PKR phosphorylation, can trigger the induction of transcription factor 4 (ATF4), and can lead to apoptosis [46]. Another study demonstrated that tunicamycin or Aβ treatment can induce PKR in human neuroblastoma cells and can trigger GSK3β activation, as well as tau phosphorylation. The pretreatment of cell cultures with the PKR inhibitor PRI peptide reduced GSK3β and PKR activation, as well as tau phosphorylation, which suggests that PKR can indirectly control GSK3β activation [47]. These results could partially explain the histological co-localization of neuronal PKR and tau detected in AD brains and could implicate PKR in signaling pathways that lead to tau phosphorylation.

## PKR and memory

AD is very often marked by initial memory disturbances, and patients are often followed over the course of the disease with memory tests such as the free and cued selective reminding test [48]. Several experimental reports have shown that the activation of PKR signaling could be associated with decreased memory performance. Previous studies have shown that local protein synthesis at synapses is required for long-lasting strength induced by, for example, BDNF [49]. Inhibitors of protein synthesis have been used to experimentally induce amnesia.

Recent studies focusing on the role of PKR and its capacity to reduce protein translation have demonstrated that a link exists between the modulation of PKR activity and memory formation. An initial study was conducted using transgenic mice only expressing inducible PKR in the hippocampal CA1 region after intra-ventricular injection of the compound AP20187 [50]. This treatment increased the expression of phosphorylated eIF2α and ATF4 and reduced CREB pathways in hippocampal neurons of the CA1 region. Under these conditions, late-phase long-term potentiation (LTP) and memory consolidation for avoidance test and fear-conditioning evaluations were reduced in treated animals. The administration of the general translation inhibitor anisomycin did not reproduce these results, which suggests that the modulation of specific genes via the PKR/eIF2α pathway was more likely to be involved than the general repression of global translation. This is an elegant demonstration of the role of PKR in experimental memory consolidation.

In 2011, Zhu et al. [51] confirmed the involvement of PKR in the molecular process of memory. They found that, in PKR knockout mice, LTP learning and memory tests were enhanced compared with control mice. These effects were associated with augmented network excitability. In addition, they demonstrated that the lack of PKR reduced the action of interferon-γ on GABAergic synapses. These cognitive improvements were reproduced after intra-peritoneal administration of the PKR inhibitor C16 in wild-type mice.

Another study has confirmed the involvement of PKR in cortex taste memory [52]. Using novel taste learning and conditioned test aversion, the authors showed that systemic or local injection of PKRi in the gustatory cortex enhanced cognitive performance in rats. These findings were associated with a reduction in PKR activation and eIF2α phosphorylation. Recent data have shown that a molecule called ISRIB (integrated stress response inhibitor) acting between eIF2α and eIF2B [53] in the process of general translation was able to increase spatial memory and fear-conditioning tests in treated animals. These results led to the conclusion that the downstream effects of ISRIB on the initial translation phase that occurs after eIF2α phosphorylation might also modulate the molecular process of memory. Surprisingly, this result was not found in another study in AD J20 transgenic mice or in wild-type mice treated with ISRIB [54]. Regarding these data, it is possible that PKR might act on memory consolidation through other pathways either downstream of or not dependent on eIF2α. Another explanation is that the disturbing cognitive effects linked to brain amyloid accumulation in these mice were too high to be compensated for by ISRIB.

The allele E4 of the apolipoprotein E gene is a major genetic risk factor for AD [55]. Overexpressing the human apolipoprotein allele E4 in transgenic mice induced increased brain eIF2α phosphorylation and abnormal learning [56]. These authors have recently shown that the injection of PKRi rescued memory deficits and decreased ATF4 expression in treated mice [57]. The authors propose that ApoE4 overexpression could modulate the PKR pathway and that PKR inhibition could restore memory impairment in the initial stages of AD. A more recent article has revealed that the phenolic glucoside gastronidin acts as a PKR inhibitor in the AD transgenic Tg2576 mouse and can reduce memory disturbances and decrease BACE1 expression in treated mice [58]. Further research is needed to decipher the exact role of PKR in memory and synaptic functions in physiological and pathological conditions.

## PKR and insulin signaling pathways

Recent studies have supported the possibility that AD could be a form of type III diabetes in which insulin resistance could play a major role [59]. Clinical trials are underway to test whether the administration of intranasal insulin could modulate cognitive decline in AD [60]. An experimental report has shown that PKR can phosphorylate insulin receptor substrate 1 (IRS1), which is a cellular event linked to insulin resistance in peripheral organs [61]. In addition, high glucose can disturb insulin signaling through the activation of PKR in muscle cells [62]. Increased apoptosis and production of ROS can be reduced by pharmacological inhibition of PKR. Another study has shown that PKR can control insulin sensitivity under physiological conditions in normal experimental animals, as well as in obese mice. The authors showed improvements in insulin sensitivity and glucose tolerance in PKR knockout mice [63]. The activation of PKR can also decrease the proliferation of pancreatic β cells once this kinase is triggered by lipotoxicity of pro-inflammatory cytokines. Cell proliferation is stopped at the G1 phase [64]. To determine whether PKR activation has a similar detrimental function in human brains will require appropriate research in AD patients. It is possible to assume that the various neuronal stresses associated with increased levels of PKR activation detected in AD brains could interfere with neuronal insulin signaling, as observed in the cells of peripheral organs during metabolic stress.

## PKR in AD patients

### Neuropathological studies

**Fig. 2:**
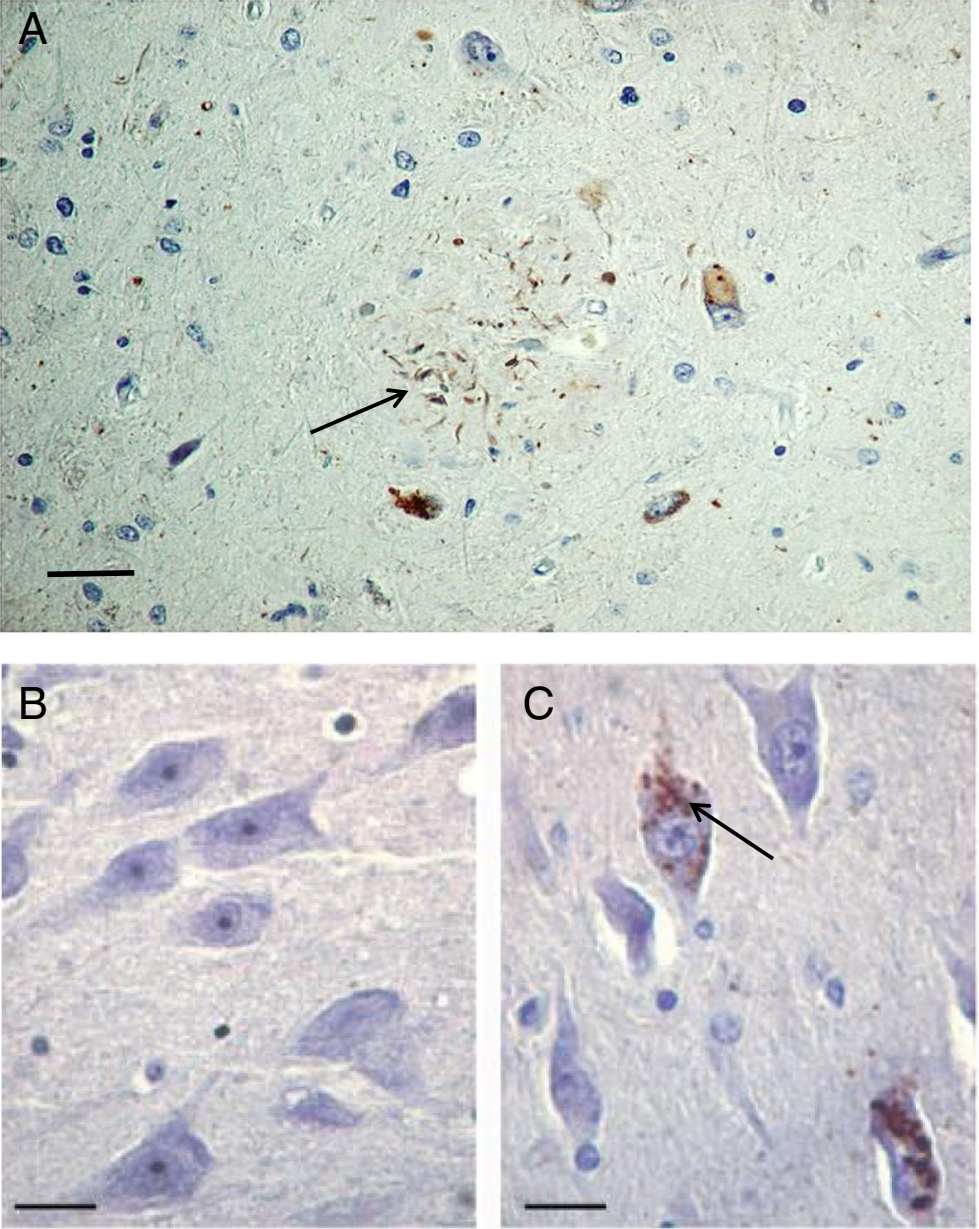
Phosphorylated PKR immunostaining in AD and control brains. **a** AD brain PKR staining is localized to neuritic dendrites surrounding an amyloid plaque, as well as in the cytosol of neighboring neurons (*bar* = 60 μm). The *arrow* indicates dystrophic neurites positive for pPKR staining. **b** No neuronal staining is depicted in a control brain (*bar* = 20 μm). **c** AD brain PKR immunostaining is seen in neuronal cytosolic vacuoles (*bar* = 20 μm). The *arrow* indicates pPKR accumulation in neurons from an AD brain. Original figure from authors

In 2002, the first report showing a link between PKR and AD revealed that degenerating neurons in the hippocampus and the frontal cortex of AD patients displayed marked immunohistochemical positivity for phosphorylated PKR and eIF2α [45] (Fig. 2). In addition, many of these neurons were also immunostained with an antibody for phosphorylated tau protein [45]. It was concluded that the PKR-eIF2α pro-apoptotic pathway could be involved in neuronal degeneration. Previously, a comparable finding was also made for Huntington’s disease [65]. These results have been confirmed in AD by new reports showing that phosphorylated PKR immunoreactivity was predominantly granular in neurons and was associated with neuritic plaques in the hippocampus and the cortex [38, 39]. Onuki et al. [32] also revealed that phosphorylated PKR was present in neurons in AD brains. In 2005, the same team showed that phosphorylated PKR could accumulate in hippocampal neurons of Parkinson’s disease (PD) and Huntington’s disease (HD) patients, and they found increased levels of this protein in hippocampal samples using western blots [66]. Regarding these findings, PKR was proposed as a putative therapeutic target to attenuate neuronal demise in these disorders [67]. Interestingly, Paquet et al also revealed that phosphorylated PKR was present in degenerative neurons in Creutzfeldt–Jakob disease and that these accumulations were correlated with the extent of neuronal apoptosis, spongiosis and microglial activation [68].

The molecular mechanism of neuronal PKR activation in AD is not known, and researchers have tried to determine whether the PKR activator PACT is involved in this process. Using immunohistochemical techniques, Paquet et al. have shown that PACT and phosphorylated PKR co-localized in degenerating neurons in AD brains and in APP/PS1 transgenic mice [69]. PACT shRNA treatment of human neuroblastoma cells decreased PKR activation produced by Aβ1-42 exposure. These results correlate with the idea that Aβ1-42 toxicity could increase PACT levels and induce PKR activation, as well as neuronal degeneration. Although abnormal neurons in AD brains could accumulate phosphorylated tau and PKR, a link between the two proteins has not been determined. In 2010, Bose et al revealed that, in AD brains, neuronal phosphorylated PKR could co-localize with activated GSK3β, which is a potent kinase that can phosphorylate tau protein [47]. A more recent histological study has shown that the loads of two pro-inflammatory kinases, PKR and JNK, had a negative correlation with cognitive scores in a cohort of 299 AD and non-AD brains [70].

Altogether, these findings argue in favor of a detrimental role for neuronal PKR activation in neurodegenerative diseases, although the exact cause of this molecular process might be different in neurons from AD, PD, or HD patients. These data indicate that triggers other than Aβ peptide can activate PKR in PD or HD, as well as perhaps in AD. This abnormal signaling, including PKR activation, could represent an initial neurodegenerative event that leads to various neuropathological lesions as a function of neuronal susceptibility.

### Blood studies

Several studies have assessed the levels of PKR in blood and cerebrospinal fluid (CSF) of AD patients and controls. An analysis of the concentrations of total and phosphorylated PKR in peripheral blood mononuclear cells (PBMCs) was carried out to assess whether PKR is a possible blood biomarker [71]. The results in 23 AD patients and 19 control individuals showed statistically significant increased levels of the ratio of PKR/phosphorylated PKR in AD patients compared with controls. These ratios were correlated with the levels of Mini Mental Status Examination (MMSE), as well as free and cued selective reminding tests in AD patients. Unfortunately, overlapping concentrations were found that did not clearly differentiate affected people from non-affected people at the individual level. Subsequent data from PBMCs have demonstrated that the concentrations of p53 and Redd1 mRNA and proteins were enhanced in AD patients and were linked to PKR levels. The conclusion of this study was that the activation of a PKR-p53-redd1 pathway could participate in translation deregulation [72]. It has been demonstrated that experimental peripheral inflammation controlled by PKR could modulate central neuroinflammation and Aβ production [43]. A research report has shown that pharmacological inhibition of PKR in PBMCs from AD patients could reduce the release of TNFα, IL-1α, and IL-6 while also preventing the activation of caspase 3. The authors indicate that peripheral inhibition of PKR might modulate the brain inflammation observed in AD [35].

The allele E4 of the apolipoprotein gene is a risk factor for AD and might also trigger the early onset of the disease. Badia et al. [73] studied gene and protein expression in PBMCs of 50 healthy subjects, with 33 subjects carrying at least one ApoE4 allele. The findings revealed that RCAN1 (regulator of calcineurin), calcineurin, and PKR mRNA levels were increased in subjects with at least one ApoE4 allele compared with subjects without any ApoE4 alleles [73]. The links between the E4 allele and PKR are not yet known but could explain the increased levels of the PKR protein in PBMCs of AD patients that were detected in early reports. Another genetic study linking the PKR gene to AD was published by Bullido et al. [74]. These authors reported that a PKR SNP (rs2254958) located in the 5′ UTR region within an exonic slicing enhancer was associated with AD. The C allele was more frequently found than the non-CC genotype in AD patients [74]. For the authors, one possible explanation for these findings was that variants of human genes involved in HSV-1 infection could modulate susceptibility to AD. In conclusion, it is certainly worth further exploring the reasons why PKR gene expression is differently regulated in AD PBMCs, and this result could be used in the future as a possible biomarker for early diagnosis and prevention trials.

### CSF studies

The CSF seems to be one of the most reliable sources of metabolic information and anomalies occurring in the brain. So far, assessments of CSF biomarkers, such as Aβ1-42, Aβ1-40, tau, and phosphorylated tau, have been widely used in clinical research and in routine practice to determine whether patients have CSF abnormalities that reflect AD brain lesions [75]. Based on results showing increased levels of PKR in AD brains, we have evaluated the CSF levels of total and phosphorylated PKR in AD patients (n = 45) in mild cognitive impairment (MCI) due to AD with abnormal CSF biomarkers (n = 11) and in neurological controls (n = 35) with normal CSF biomarkers [76]. The findings revealed significantly augmented concentrations of total and phosphorylated PKR in AD and MCI patients compared with neurological controls. The sensitivity was 91.1% and the specificity was 94.3%. Very few overlapping results were observed. CSF PKR levels correlated with phosphorylated tau levels. In a subsequent study, affected individuals were followed for 2 years with repeated cognitive evaluations. Patients with high levels of CSF phosphorylated PKR had a more rapid cognitive decline than patients with low CSF phosphorylated PKR concentrations [77]. Other CSF biomarkers were not associated with cognitive deterioration during the follow-up period. The development of reliable tests to measure CSF PKR levels and other kinases could bring about new useful biomarkers that could facilitate the diagnosis of early AD brain lesions, as well as provide a possible prediction for future cognitive decline in patients with dementia and in non-dementia patients [78].

## Pharmacological interventions

As reported previously in this review, several studies have used PKR genetic blockade or PKR pharmacological inhibition to modulate the molecular process of memory formation or AD brain lesions in experimental models and transgenic AD mice. Unfortunately, no pharmacological PKR inhibitors have reached clinical phase 1 or subsequent phases of clinical trials. The consequences of the active anti-amyloid therapy AN 1792 on brain PKR and tau loads have recently been studied [79]. In non-immunized patients, the magnitude of axonal degradation (neuritic curvature ratio) and spongiosis was correlated with the levels of phosphorylated PKR load assessed by immunohistochemical methods. In immunized patients, the reduction of PKR load was associated with Aβ1-42 removal and the decrease of microglial markers. These results underlined the links between Aβ1-42 accumulation, PKR activation, and neuroinflammation.

## Conclusions

The initial cause of AD is unknown, and the trigger for inducing the accumulation of Aβ oligomers in sporadic AD is also unknown. Whether activation of PKR in AD follows the accumulation of Aβ or is located upstream of this amyloid pathway and leads to BACE 1 induction has not yet been determined. PKR can be activated by so many stresses that lead to ISR that an association with subtle brain inflammation (viral or infectious), ER or oxidative stress, and metabolic abnormalities could increase BACE 1 translation and Aβ synthesis. These events, which may be associated, for example, with an infection, trauma, or an unknown aging process, could occur decades before the first clinical signs and may be reinforced by Aβ oligomer production. As mentioned before, brain PKR activation is independent of Aβ accumulation in PD and HD and could be linked to α-synuclein and abnormal metabolism of huntingtin. It is plausible that once the neurotoxic cascade is switched on, several abnormal molecular pathways could contribute to this vicious circle and lead to AD through tau phosphorylation, synaptic degradation, and initial memory disturbances. A comparable cascade involving other proteins could theoretically be proposed for other neurodegenerative disorders, such as PD and HD. In the future, early pharmacological inhibition of kinases associated with a reduction of Aβ oligomer synthesis might support efficient multi-target therapy. The discovery of new PKR inhibitors seems to be an appropriate goal for a new therapeutic approach, especially if the sum of the early initial brain cellular events can contribute to the activation of PKR and other potential toxic kinases. In addition, new biological methods detecting subtle PKR anomalies in the blood and/or in the CSF in pre-symptomatic or prodromal AD patients could facilitate the validation of target engagement. Early detection and treatment of AD brain lesions, including PKR deregulation, might provide a sensitive way to put in place secondary prevention to reduce the relentless burden on patients and their caregivers.

## Abbreviations

AD: Alzheimer’s disease
APP: Amyloid precursor protein
CSF: Cerebrospinal fluid
eIF2α: Eukaryotic initiation factor 2α
ER: Endoplasmic reticulum
HD: Huntington’s disease
IL: Interleukin
ISR: Integrated stress response
LTP: Long-term potentiation
MCI: Mild cognitive impairment
PACT: PKR activator
PBMC: Peripheral blood mononuclear cell
PD: Parkinson’s disease
PKR: Eukaryotic initiation factor 2 kinase 2
PKRi: PKR inhibitor
ROS: Reactive oxygen species
TNF: Tumor necrosis factor
UPR: Unfolded protein response

## References

[1] Scheltens P, Blennow K, Breteler MM, de Strooper B, Frisoni GB, Salloway S, Van der Flier WM (2016). Alzheimer’s disease. Lancet.

[2] Montine TJ, Phelps CH, Beach TG, Bigio EH, Cairns NJ, Dickson DW, Duyckaerts C, Frosch MP, Masliah E, Mirra SS (2012). National Institute on Aging-Alzheimer’s Association guidelines for the neuropathologic assessment of Alzheimer’s disease: a practical approach. Acta Neuropathol.

[3] Selkoe DJ, Hardy J (2016). The amyloid hypothesis of Alzheimer’s disease at 25 years. EMBO Mol Med.

[4] Hardy J, Bogdanovic N, Winblad B, Portelius E, Andreasen N, Cedazo-Minguez A, Zetterberg H (2014). Pathways to Alzheimer’s disease. J Intern Med.

[5] Iqbal K, Liu F, Gong CX (2016). Tau and neurodegenerative disease: the story so far. Nat Rev Neurol.

[6] Medina M, Avila J (2015). Further understanding of tau phosphorylation: implications for therapy. Expert Rev Neurother.

[7] Tell V, Hilbrich I, Holzer M, Totzke F, Schachtele C, Slynko I, Sippl W, Hilgeroth A (2016). drug development of small-molecule inhibitors of AD-relevant kinases as novel perspective multitargeted approach. Curr Alzheimer Res.

[8] Garcia MA, Meurs EF, Esteban M (2007). The dsRNA protein kinase PKR: virus and cell control. Biochimie.

[9] Rice AP, Duncan R, Hershey JW, Kerr IM (1985). Double-stranded RNA-dependent protein kinase and 2-5A system are both activated in interferon-treated, encephalomyocarditis virus-infected HeLa cells. J Virol.

[10] Pakos-Zebrucka K, Koryga I, Mnich K, Ljujic M, Samali A, Gorman AM (2016). The integrated stress response. EMBO Rep.

[11] Chen A, Muzzio IA, Malleret G, Bartsch D, Verbitsky M, Pavlidis P, Yonan AL, Vronskaya S, Grody MB, Cepeda I (2003). Inducible enhancement of memory storage and synaptic plasticity in transgenic mice expressing an inhibitor of ATF4 (CREB-2) and C/EBP proteins. Neuron.

[12] Pasini S, Corona C, Liu J, Greene LA, Shelanski ML (2015). Specific downregulation of hippocampal ATF4 reveals a necessary role in synaptic plasticity and memory. Cell Rep.

[13] Scheper W, Hoozemans JJ (2015). The unfolded protein response in neurodegenerative diseases: a neuropathological perspective. Acta Neuropathol.

[14] Halliday M, Mallucci GR (2015). Review: Modulating the unfolded protein response to prevent neurodegeneration and enhance memory. Neuropathol Appl Neurobiol.

[15] Rozpedek W, Markiewicz L, Diehl JA, Pytel D, Majsterek I (2015). Unfolded protein response and PERK kinase as a new therapeutic target in the pathogenesis of Alzheimer’s disease. Curr Med Chem.

[16] Shimazawa M, Hara H (2006). Inhibitor of double stranded RNA-dependent protein kinase protects against cell damage induced by ER stress. Neurosci Lett.

[17] Shimazawa M, Ito Y, Inokuchi Y, Hara H (2007). Involvement of double-stranded RNA-dependent protein kinase in ER stress-induced retinal neuron damage. Invest Ophthalmol Vis Sci.

[18] Alirezaei M, Watry DD, Flynn CF, Kiosses WB, Masliah E, Williams BR, Kaul M, Lipton SA, Fox HS (2007). Human immunodeficiency virus-1/surface glycoprotein 120 induces apoptosis through RNA-activated protein kinase signaling in neurons. J Neurosci.

[19] Dedoni S, Olianas MC, Onali P (2010). Interferon-beta induces apoptosis in human SH-SY5Y neuroblastoma cells through activation of JAK-STAT signaling and down-regulation of PI3K/Akt pathway. J Neurochem.

[20] Tronel C, Page G, Bodard S, Chalon S, Antier D (2014). The specific PKR inhibitor C16 prevents apoptosis and IL-1beta production in an acute excitotoxic rat model with a neuroinflammatory component. Neurochem Int.

[21] Shen S, Niso-Santano M, Adjemian S, Takehara T, Malik SA, Minoux H, Souquere S, Marino G, Lachkar S, Senovilla L (2012). Cytoplasmic STAT3 represses autophagy by inhibiting PKR activity. Mol Cell.

[22] Bordi M, Berg MJ, Mohan PS, Peterhoff CM, Alldred MJ, Che S, Ginsberg SD, Nixon RA (2016). Autophagy flux in CA1 neurons of Alzheimer hippocampus: Increased induction overburdens failing lysosomes to propel neuritic dystrophy. Autophagy.

[23] Kang R, Tang D (2012). PKR-dependent inflammatory signals. Sci Signal.

[24] Lu B, Nakamura T, Inouye K, Li J, Tang Y, Lundback P, Valdes-Ferrer SI, Olofsson PS, Kalb T, Roth J (2012). Novel role of PKR in inflammasome activation and HMGB1 release. Nature.

[25] Boriushkin E, Wang JJ, Li J, Bhatta M, Zhang SX (2016). p58(IPK) suppresses NLRP3 inflammasome activation and IL-1beta production via inhibition of PKR in macrophages. Sci Rep.

[26] Yim HC, Wang D, Yu L, White CL, Faber PW, Williams BR, Sadler AJ (2016). The kinase activity of PKR represses inflammasome activity. Cell Res.

[27] Bonnet MC, Weil R, Dam E, Hovanessian AG, Meurs EF (2000). PKR stimulates NF-kappaB irrespective of its kinase function by interacting with the IkappaB kinase complex. Mol Cell Biol.

[28] Goh KC (2000). deVeer MJ, Williams BR. The protein kinase PKR is required for p38 MAPK activation and the innate immune response to bacterial endotoxin. EMBO J.

[29] Chang RC, Suen KC, Ma CH, Elyaman W, Ng HK, Hugon J (2002). Involvement of double-stranded RNA-dependent protein kinase and phosphorylation of eukaryotic initiation factor-2alpha in neuronal degeneration. J Neurochem.

[30] Suen KC, Yu MS, So KF, Chang RC, Hugon J (2003). Upstream signaling pathways leading to the activation of double-stranded RNA-dependent serine/threonine protein kinase in beta-amyloid peptide neurotoxicity. J Biol Chem.

[31] Yu MS, Suen KC, Kwok NS, So KF, Hugon J, Chang RC (2006). Beta-amyloid peptides induces neuronal apoptosis via a mechanism independent of unfolded protein responses. Apoptosis.

[32] Onuki R, Bando Y, Suyama E, Katayama T, Kawasaki H, Baba T, Tohyama M, Taira K (2004). An RNA-dependent protein kinase is involved in tunicamycin-induced apoptosis and Alzheimer’s disease. EMBO J.

[33] Vaughn LS, Snee B, Patel RC (2014). Inhibition of PKR protects against tunicamycin-induced apoptosis in neuroblastoma cells. Gene.

[34] Gourmaud S, Mouton-Liger F, Abadie C, Meurs EF, Paquet C, Hugon J (2016). Dual kinase inhibition affords extended in vitro neuroprotection in amyloid-beta toxicity. J Alzheimers Dis.

[35] Couturier J, Page G, Morel M, Gontier C, Claude J, Pontcharraud R, Fauconneau B, Paccalin M (2010). Inhibition of double-stranded RNA-dependent protein kinase strongly decreases cytokine production and release in peripheral blood mononuclear cells from patients with Alzheimer’s disease. J Alzheimers Dis.

[36] Mouton-Liger F, Paquet C, Dumurgier J, Bouras C, Pradier L, Gray F, Hugon J (2012). Oxidative stress increases BACE1 protein levels through activation of the PKR-eIF2alpha pathway. Biochim Biophys Acta.

[37] Kim S, Sato Y, Mohan PS, Peterhoff C, Pensalfini A, Rigoglioso A, Jiang Y, Nixon RA (2016). Evidence that the rab5 effector APPL1 mediates APP-betaCTF-induced dysfunction of endosomes in Down syndrome and Alzheimer’s disease. Mol Psychiatry.

[38] Peel AL (2004). PKR activation in neurodegenerative disease. J Neuropathol Exp Neurol.

[39] Peel AL, Bredesen DE (2003). Activation of the cell stress kinase PKR in Alzheimer’s disease and human amyloid precursor protein transgenic mice. Neurobiol Dis.

[40] Page G, Rioux Bilan A, Ingrand S, Lafay-Chebassier C, Pain S, Perault Pochat MC, Bouras C, Bayer T, Hugon J (2006). Activated double-stranded RNA-dependent protein kinase and neuronal death in models of Alzheimer’s disease. Neuroscience.

[41] Lourenco MV, Clarke JR, Frozza RL, Bomfim TR, Forny-Germano L, Batista AF, Sathler LB, Brito-Moreira J, Amaral OB, Silva CA (2013). TNF-alpha mediates PKR-dependent memory impairment and brain IRS-1 inhibition induced by Alzheimer’s beta-amyloid oligomers in mice and monkeys. Cell Metab.

[42] Mouton-Liger F, Rebillat AS, Gourmaud S, Paquet C, Leguen A, Dumurgier J, Bernadelli P, Taupin V, Pradier L, Rooney T (2015). PKR downregulation prevents neurodegeneration and beta-amyloid production in a thiamine-deficient model. Cell Death Dis.

[43] Carret-Rebillat AS, Pace C, Gourmaud S, Ravasi L, Montagne-Stora S, Longueville S, Tible M, Sudol E, Chang RC, Paquet C (2015). Neuroinflammation and Abeta accumulation linked to systemic inflammation are decreased by genetic PKR down-regulation. Sci Rep.

[44] Couturier J, Paccalin M, Lafay-Chebassier C, Chalon S, Ingrand I, Pinguet J, Pontcharraud R, Guillard O, Fauconneau B, Page G (2012). Pharmacological inhibition of PKR in APPswePS1dE9 mice transiently prevents inflammation at 12 months of age but increases Abeta42 levels in the late stages of the Alzheimer’s disease. Curr Alzheimer Res.

[45] Chang RC, Wong AK, Ng HK, Hugon J (2002). Phosphorylation of eukaryotic initiation factor-2alpha (eIF2alpha) is associated with neuronal degeneration in Alzheimer’s disease. Neuroreport.

[46] Kim SM, Yoon SY, Choi JE, Park JS, Choi JM, Nguyen T, Kim DH (2010). Activation of eukaryotic initiation factor-2 alpha-kinases in okadaic acid-treated neurons. Neuroscience.

[47] Bose A, Mouton-Liger F, Paquet C, Mazot P, Vigny M, Gray F, Hugon J (2011). Modulation of tau phosphorylation by the kinase PKR: implications in Alzheimer’s disease. Brain Pathol.

[48] Grober E, Buschke H, Crystal H, Bang S, Dresner R (1988). Screening for dementia by memory testing. Neurology.

[49] Trinh MA, Klann E (2013). Translational control by eIF2alpha kinases in long-lasting synaptic plasticity and long-term memory. Neurobiol Learn Mem.

[50] Jiang Z, Belforte JE, Lu Y, Yabe Y, Pickel J, Smith CB, Je HS, Lu B, Nakazawa K (2010). eIF2alpha Phosphorylation-dependent translation in CA1 pyramidal cells impairs hippocampal memory consolidation without affecting general translation. J Neurosci.

[51] Zhu PJ, Huang W, Kalikulov D, Yoo JW, Placzek AN, Stoica L, Zhou H, Bell JC, Friedlander MJ, Krnjevic K (2011). Suppression of PKR promotes network excitability and enhanced cognition by interferon-gamma-mediated disinhibition. Cell.

[52] Stern E, Chinnakkaruppan A, David O, Sonenberg N, Rosenblum K (2013). Blocking the eIF2alpha kinase (PKR) enhances positive and negative forms of cortex-dependent taste memory. J Neurosci.

[53] Sekine Y, Zyryanova A, Crespillo-Casado A, Fischer PM, Harding HP, Ron D (2015). Stress responses. Mutations in a translation initiation factor identify the target of a memory-enhancing compound. Science.

[54] Johnson EC, Kang J (2016). A small molecule targeting protein translation does not rescue spatial learning and memory deficits in the hAPP-J20 mouse model of Alzheimer’s disease. PeerJ.

[55] Strittmatter WJ, Saunders AM, Schmechel D, Pericak-Vance M, Enghild J, Salvesen GS, Roses AD (1993). Apolipoprotein E: high-avidity binding to beta-amyloid and increased frequency of type 4 allele in late-onset familial Alzheimer disease. Proc Natl Acad Sci U S A.

[56] Segev Y, Barrera I, Ounallah-Saad H, Wibrand K, Sporild I, Livne A, Rosenberg T, David O, Mints M, Bramham CR (2015). PKR inhibition rescues memory deficit and ATF4 overexpression in ApoE epsilon4 human replacement mice. J Neurosci.

[57] Segev Y, Michaelson DM, Rosenblum K (2013). ApoE epsilon4 is associated with eIF2alpha phosphorylation and impaired learning in young mice. Neurobiol Aging.

[58] Zhang JS, Zhou SF, Wang Q, Guo JN, Liang HM, Deng JB, He WY (2016). Gastrodin suppresses BACE1 expression under oxidative stress condition via inhibition of the PKR/eIF2alpha pathway in Alzheimer’s disease. Neuroscience.

[59] Diehl T, Mullins R, Kapogiannis D (2017). Insulin resistance in Alzheimer’s disease. Transl Res.

[60] Claxton A, Baker LD, Hanson A, Trittschuh EH, Cholerton B, Morgan A, Callaghan M, Arbuckle M, Behl C, Craft S (2015). Long acting intranasal insulin detemir improves cognition for adults with mild cognitive impairment or early-stage Alzheimer’s disease dementia. J Alzheimers Dis.

[61] Nakamura T, Furuhashi M, Li P, Cao H, Tuncman G, Sonenberg N, Gorgun CZ, Hotamisligil GS (2010). Double-stranded RNA-dependent protein kinase links pathogen sensing with stress and metabolic homeostasis. Cell.

[62] Udumula MP, Babu MS, Bhat A, Dhar I, Sriram D, Dhar A (2017). High glucose impairs insulin signaling via activation of PKR pathway in L6 muscle cells. Biochem Biophys Res Commun.

[63] Carvalho-Filho MA, Carvalho BM, Oliveira AG, Guadagnini D, Ueno M, Dias MM, Tsukumo DM, Hirabara SM, Reis LF, Curi R (2012). Double-stranded RNA-activated protein kinase is a key modulator of insulin sensitivity in physiological conditions and in obesity in mice. Endocrinology.

[64] Chen SS, Jiang T, Wang Y, Gu LZ, Wu HW, Tan L, Guo J (2014). Activation of double-stranded RNA-dependent protein kinase inhibits proliferation of pancreatic beta-cells. Biochem Biophys Res Commun.

[65] Peel AL, Rao RV, Cottrell BA, Hayden MR, Ellerby LM, Bredesen DE (2001). Double-stranded RNA-dependent protein kinase, PKR, binds preferentially to Huntington’s disease (HD) transcripts and is activated in HD tissue. Hum Mol Genet.

[66] Bando Y, Onuki R, Katayama T, Manabe T, Kudo T, Taira K, Tohyama M (2005). Double-strand RNA dependent protein kinase (PKR) is involved in the extrastriatal degeneration in Parkinson’s disease and Huntington’s disease. Neurochem Int.

[67] Hugon J, Paquet C, Chang RC (2009). Could PKR inhibition modulate human neurodegeneration?. Expert Rev Neurother.

[68] Paquet C, Bose A, Polivka M, Peoc’h K, Brouland JP, Keohane C, Hugon J, Gray F (2009). Neuronal phosphorylated RNA-dependent protein kinase in Creutzfeldt-Jakob disease. J Neuropathol Exp Neurol.

[69] Paquet C, Mouton-Liger F, Meurs EF, Mazot P, Bouras C, Pradier L, Gray F, Hugon J (2012). The PKR activator PACT is induced by Abeta: involvement in Alzheimer’s disease. Brain Pathol.

[70] Taga M, Minett T, Classey J, Matthews FE, Brayne C, Ince PG, Nicoll JA, Hugon J, Boche D, Mrc C (2017). Metaflammasome components in the human brain: a role in dementia with Alzheimer’s pathology?. Brain Pathol.

[71] Paccalin M, Pain-Barc S, Pluchon C, Paul C, Besson MN, Carret-Rebillat AS, Rioux-Bilan A, Gil R, Hugon J (2006). Activated mTOR and PKR kinases in lymphocytes correlate with memory and cognitive decline in Alzheimer’s disease. Dement Geriatr Cogn Disord.

[72] Damjanac M, Page G, Ragot S, Laborie G, Gil R, Hugon J, Paccalin M (2009). PKR, a cognitive decline biomarker, can regulate translation via two consecutive molecular targets p53 and Redd1 in lymphocytes of AD patients. J Cell Mol Med.

[73] Badia MC, Lloret A, Giraldo E, Dasi F, Olaso G, Alonso MD, Vina J (2013). Lymphocytes from young healthy persons carrying the ApoE4 allele overexpress stress-related proteins involved in the pathophysiology of Alzheimer’s disease. J Alzheimers Dis.

[74] Bullido MJ, Martinez-Garcia A, Tenorio R, Sastre I, Munoz DG, Frank A, Valdivieso F (2008). Double stranded RNA activated EIF2 alpha kinase (EIF2AK2; PKR) is associated with Alzheimer’s disease. Neurobiol Aging.

[75] Lista S, O’Bryant SE, Blennow K, Dubois B, Hugon J, Zetterberg H, Hampel H (2015). Biomarkers in sporadic and familial Alzheimer’s disease. J Alzheimers Dis.

[76] Mouton-Liger F, Paquet C, Dumurgier J, Lapalus P, Gray F, Laplanche JL, Hugon J (2012). Groupe d’Investigation du Liquide Cephalorachidien Study N. Increased cerebrospinal fluid levels of double-stranded RNA-dependant protein kinase in Alzheimer’s disease. Biol Psychiatry.

[77] Dumurgier J, Mouton-Liger F, Lapalus P, Prevot M, Laplanche JL, Hugon J, Paquet C, Groupe d’Investigation du Liquide Cephalorachidien Study N (2013). Cerebrospinal fluid PKR level predicts cognitive decline in Alzheimer’s disease. PLoS One.

[78] Paquet C, Dumurgier J, Hugon J (2015). Pro-apoptotic kinase levels in cerebrospinal fluid as potential future biomarkers in Alzheimer’s disease. Front Neurol.

[79] Paquet C, Amin J, Mouton-Liger F, Nasser M, Love S, Gray F, Pickering RM, Nicoll JA, Holmes C, Hugon J (2015). Effect of active Abeta immunotherapy on neurons in human Alzheimer’s disease. J Pathol.

